# *Ex Vivo* Tumor-Derived Organoid Pharmacotyping Identifies Personalized Therapeutic Options for Patients with Biliary Tract Cancer

**DOI:** 10.1158/2767-9764.CRC-25-0792

**Published:** 2026-08-31

**Authors:** Annie Blair Richardson, Payel Chatterjee, Rachele Rosati, Alex Rajewski, Robert L. Diaz, Lauren R. Appleyard, Shalini Pereira, Brady Bernard, Milind M. Javle, Gentry G. King, Adam Diehl, William Proctor Harris, Sameek Roychowdhury, Christopher J. Kemp, Carla Grandori

**Affiliations:** 1 Cure First, Bothell, Washington.; 2 Tempus AI, Chicago, Illinois.; 3 Providence Cancer Institute, Portland, Oregon.; 4 The University of Texas MD Anderson Cancer Center, Houston, Texas.; 5 Department of Gastrointestinal Medical Oncology, University of Washington School of Medicine, Seattle, Washington.; 6 Ohio State University Comprehensive Cancer Care Center, Columbus, Ohio.; 7 Human Biology Division, Fred Hutchinson Cancer Center, Seattle, Washington.

## Abstract

Biliary tract cancers (BTC) pose clinical challenges due to poor chemotherapy response and aggressive disease course. We evaluated patient-derived tumor organoid–based drug sensitivity testing as a tool to guide therapy. In this multicenter study, 26 tumor organoids were successfully derived from 43 patients with BTC and tested with an average of 50 cancer-directed therapies using the Clinical Laboratory Improvement Amendments–certified PARIS assay. Despite most organoids being from late-stage disease, 24/26 (92.3%) exhibited strong sensitivity to one or more targeted agents. Active drugs included inhibitors of EGFR/HER2, MEK, ERK, BCR-ABL and SRC family, mTOR, PI3K, MDM2, BCL2, and BET. Drug sensitivities aligned with known genetic biomarkers but were also observed in cultures lacking them, indicating *ex vivo* testing can expand actionability beyond genomics. In five cases, results guided therapy; one patient with an *FGFR-BICC1* fusion refractory to FGFR inhibitors responded to dasatinib, achieving symptomatic improvement, stable disease, and >8-month survival.

**Significance:**

*Ex vivo* drug testing of tumor-derived organoids is clinically feasible and can be used to identify personalized treatment options for patients with BTC, to evaluate the functional relevance of genomic biomarkers, and to guide treatment in real time.

## Introduction

Biliary tract cancer (BTC) is the second most common primary liver cancer, with an estimated 12,000 new cases per year in the United States (1, 2). BTCs are heterogeneous epithelial neoplasms and are subdivided into gallbladder cancer, intrahepatic cholangiocarcinoma (iCCA), extrahepatic cholangiocarcinoma (eCCA), and combined hepatocellular cholangiocarcinoma (HCC-CCA), each of which has distinct genetic profiles, biology, and clinical features (3, 4). Approximately 60% to 70% of patients present with inoperable or metastatic disease, and there is a high relapse rate in patients who undergo surgery (5).

The preferred first-line therapy for advanced BTC in patients eligible for intensive systemic therapy includes combinations of cisplatin, gemcitabine, and either durvalumab or pembrolizumab (National Comprehensive Cancer Network guidelines). Second-line systemic therapy for patients lacking targetable genomic alterations includes folinic acid, fluorouracil and oxaliplatin (FOLFOX; ABC-06) and 5-fluorouracil/nanoliposomal irinotecan. However, response rates and survival with all of these regimens are poor (6, 7), and further treatment selection strategies are lacking. The median overall survival for patients with BTC is less than 12 months, and the 5-year survival is less than 18% (8), highlighting the pressing need to identify patient-specific therapeutic options.

Recently, several biomarker-driven targeted therapies have been approved, including the FGFR inhibitors pemigatinib, futibatinib, and infigratinib for CCAs with *FGFR2* fusions or rearrangements (9–11) and the IDH1 inhibitor ivosidenib for CCAs with *IDH1* mutations (12). However, alterations in *IDH1* and *FGFR2* occur in only 4% to 15% and 5% to 12% of CCA cases, respectively (13–15), and most tumors develop resistance to these targeted therapies (16, 17). Other recurrent genetic alterations in BTCs include mutations in *TP53*, *ARID1A*, *KRAS*, *BRAF*, *BRCA1*, *BRCA2*, *MET*, amplification of *ERBB2 and ERBB3*, and loss of *CDKN2A*/*CDKN2B* (18). Targeted FDA-approved drugs are available for some of these driver mutations in other tumor types, with the potential to expand treatment options (19), but functional and clinical validation are needed to advance BTC-specific indications.

Given the limited overall survival of patients with BTC and the aggressive course of the disease, evidence-based methods of choosing additional treatment options are urgently needed. Tumor organoids, derived from individual patients with cancer, share both germline variants and somatic genomic alterations from each patient’s originating tumor (20). Patient-derived tumor organoids (PDTO) also share any prior environmental or treatment exposures of a given patient’s tumor. As these genetic and environmental variables can significantly modify drug sensitivity, functional assays using PDTOs may improve drug sensitivity prediction and accelerate biomarker evaluation. PDTOs have only recently been developed as a surrogate model system to enable *ex vivo* drug testing of a given patient’s tumor cells in a clinical setting (21–25), and very few studies have focused on BTC or applications to clinical practice (26, 27). Here, we present findings from a multicenter study that evaluated the feasibility of integrating the PARIS assay, a Clinical Laboratory Improvement Amendments (CLIA)–certified organoid-based drug sensitivity test, into the clinical management of patients with BTC. We demonstrate how this functional precision medicine assay can be used to evaluate the functional relevance of genetic biomarkers, study drug resistance in individual patients, and guide treatment selection.

## Materials and Methods

### Tissue collection

Fresh tissue samples were collected with informed written consent that complied with recognized ethical guidelines (e.g., Declaration of Helsinki, CIOMS, Belmont Report, U.S. Common Rule), and the studies were approved by an Institutional Review Board (IRB). A subset of the patients (BTC25, BTC26, BTC27, BTC29, BTC33, BTC34, BTC37, and BTC43) were under a study (A Prospective Feasibility Study of Multi-Platform Profiling Using Biospecimens from Patients with Resected Biliary Tract Cancer: UW Study: NCT04561453) sponsored by the University of Washington and consented through their IRB and related consent, which included SEngine Precision Medicine. Specimens, including surgical excisional biopsies, ascites, core needle biopsies, or punch biopsies, were shipped to arrive within 48 hours for drug testing at our CLIA laboratory.

### Generation of patient-derived BTC 3D models

BTC specimens (biopsies and surgical resections) were processed by mechanical dissociation into smaller tissue fragments, followed by enzymatic dissociation using the Miltenyi Biotec Human Tumor Dissociation Kit (cat. #130-095-929) when specimen size permitted. Liquid specimens (ascites, pleural effusion) were centrifuged to obtain cell pellets. During the initial culture phase, samples underwent enrichment to deplete red blood cells, lymphocytes, and cancer-associated fibroblasts using specialized expansion media in ultralow attachment plates. The culture medium consisted of DMEM/F-12 supplemented with HEPES, glucose, L-glutamine, Primocin (Invivogen, cat. #ant-pm-1), penicillin–streptomycin (Sigma-Aldrich), B27 (Gibco, cat. #17504-099), N-2 supplement (Thermo Fisher Scientific, #17502048), GlutaMAX (Gibco, # 35050-061), Noggin (R&D Systems, #6057-NG/CF), Wnt-3a (R&D Systems, #5036-WN/CF), R-spondin-1 (PeproTech, #120-38), nicotinamide (Sigma-Aldrich, #N0636), N-acetylcysteine (Sigma, #A9165), recombinant HGF (Millipore, #GF116), bFGF (Corning, #354060), FGF10 (PeproTech, #100-26), A83-01 (Sigma-Aldrich, #SML0788), forskolin (Selleckchem, #S2449), gastrin (Sigma-Aldrich, #G9145), rhEGF (Corning, #354052), and ROCK inhibitor (Y-27632; Selleckchem, #S1049). Enriched tumor cells or tissue progenitors were embedded in extracellular matrix scaffolds (e.g., Matrigel) and maintained under serum-free conditions. Once organoids reached approximately 200 to 500 μm in diameter, they were dissociated with trypsin-EDTA 0.25% and re-embedded in fresh Matrigel for expansion. Once the cultures reached ≥70% tumor cell content by bright field microscopy and >70% viability by Trypan blue staining, 3D cultures were subjected to high-throughput drug testing.

To establish the oncogenic identity and clinical relevance of our patient-derived CCA 3D models, we demonstrated their genotypic fidelity to the corresponding parental tumors with whole-exome sequencing (WES; conducted by CLIA-certified Fulgent Genetics), and an internal analytic pipeline was applied as described in the “WES” section.

### High-throughput drug testing

A customized panel of an average of 50 drugs for each patient was selected from a library of >200 cancer-directed therapies, based on oncologists’ requests, genetic alterations in the patient’s tumor, and the genetic landscape of BTC. The drug library consisted of FDA-approved chemotherapies and targeted drugs, as well as drugs in current clinical trials. Liquid handling robotics were used for cell plating and viability assays. Drugs were dispensed using contactless acoustic droplet ejection (Echo, Labcyte), employing in-house developed software to monitor active drug lots and tailor tests for individual patients. Cell cultures were dissociated to single cells and plated into 384-well plates on day 0 in the presence or absence of Matrigel. On day 1, drugs were added, and cells were incubated for 6 days in the presence of six different drug concentrations. On day 7, plates were read using CellTiter-Glo (Promega) and either a BioTek Synergy H4 plate reader or an EnVision plate reader (Beckman Coulter). Concentration–response curves were generated for each drug using a four-parameter logistic model. Each drug screen was subjected to quality control according to a Z’ factor derived from a strongly cytotoxic drug and negative vehicle controls.

### Scoring system to rank drug sensitivity

A proprietary algorithm was applied to each drug’s concentration–response curve to generate a numerical sensitivity score [Sengine Precision Medicine (SPM) score, range 1–15]. The score is derived from two primary parameters: (i) the solvent-normalized area under the curve, capturing the overall magnitude of drug sensitivity, and (ii) a personalization metric that benchmarks each drug response against an internal reference database of tumor responses tested with the same drug library lot.

SPM scores ≥9 are considered active; scores <9 indicate no measurable response. Active scores are grouped into descriptive categories: exceptional (15–14), good (13–12), and moderate to low (11–9). Exact threshold values and the nature of the personalization metric are proprietary and subject to a pending patent application.

Drug response was measured at a single endpoint of 6 days following drug addition. This time point was selected based on comparative assays across hundreds of drugs, demonstrating that PDTOs exhibit a broader and more informative window of response at 6 days compared with the conventional 72-hour endpoint used in standard chemosensitivity assays, better reflecting the biology of slower-cycling primary tumor cells.

### WES

Next-generation CLIA-certified WES was performed by Fulgent Genetics. Genomic DNA was extracted from tumor organoids and assessed for quality using a Bioanalyzer (Agilent Technologies). Target enrichment was then performed using the xGen Exome Research Panel v1 (Integrated DNA Technologies), followed by sequencing library preparation using the KAPA HyperPlus Library Prep kit. Indexed libraries were pooled for sequencing on multiple lanes on HiSeq (Illumina) or NovaSeq (Illumina) instruments to generate 150 bp paired-end reads at a read depth sufficient to obtain an average coverage of 300×. An in-house pipeline that follows GATK best practices for somatic short variant and insertion/deletion (indel) discovery (Broad Institute) was developed to process WES data. Briefly, short reads were mapped to the human NCBI Build 38 reference (hg38) using Burrows–Wheeler Aligner. Any potential PCR duplicates, ambiguous reads, inconsistent read pairs, or unmatched reads were excluded. Only unique reads that mapped in consistent read pairs (with proper insert size and orientation) were included for further analysis. Base substitutions and indels were called using five variant callers: FreeBayes 1.1.0, GATK3, SAMtools v1.7 (28), VarScan2 v2.4.2 (29), and VarDict v1.5.1 (30). SnpEff v4.2 (31) and AnnoVar (32, 33) variants that were present in the Genome Aggregation Database reference dataset (34) at a frequency ≥5% were considered germline variants and excluded from the analysis. Variants were retained only if they were located in RefSeq canonical transcripts, present at an allele fraction greater than 1%, and observed in more than five sequencing reads.

## Results

### Cohort of patients with BTC and tumor organoid derivation

**Figure 1.**
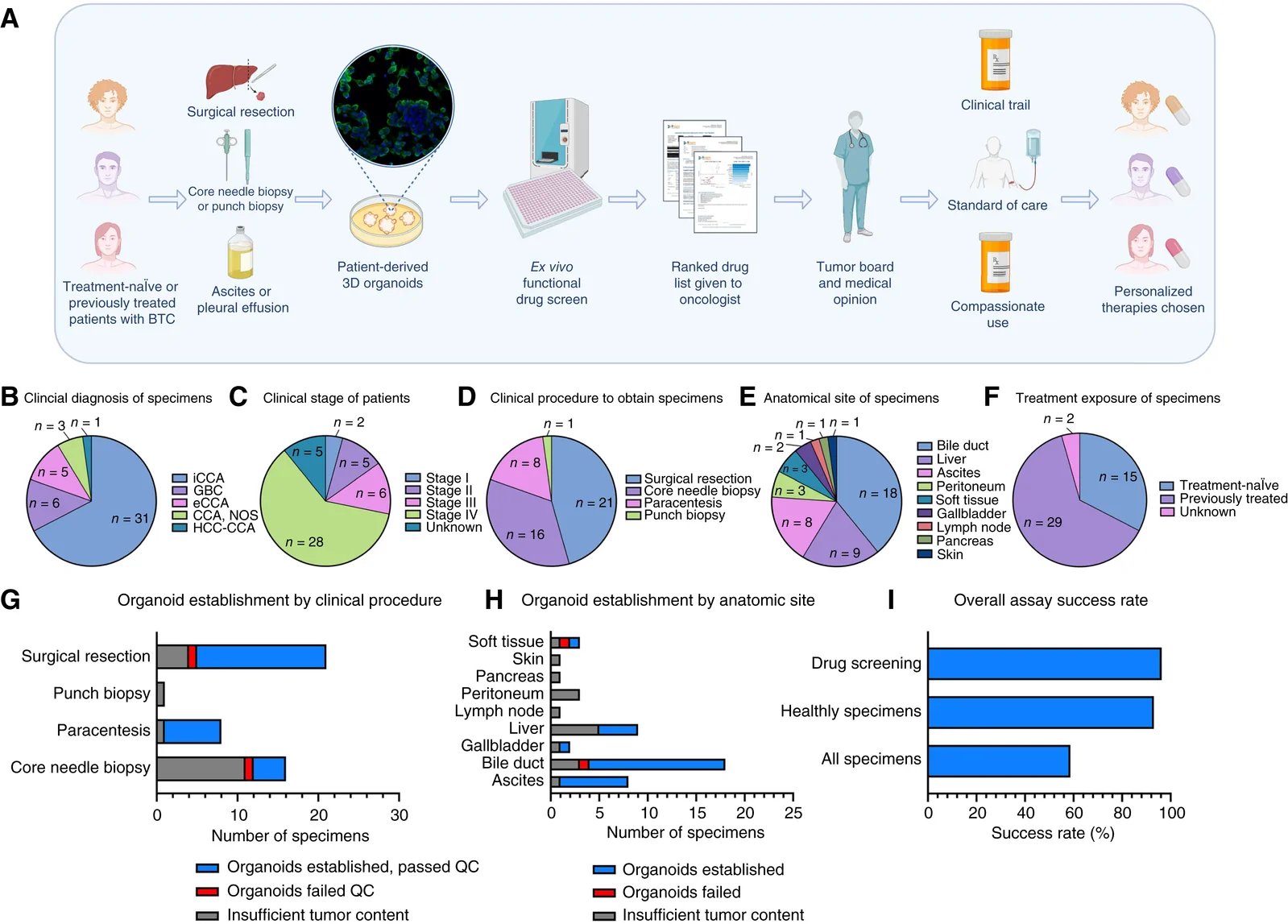
Description of PARIS assay workflow and characterization of the BTC cohort. **A,** Fresh tumor specimens from patients with BTC were sent to SEngine Precision Medicine for the derivation of organoid cultures (left). The reference organoid photo shown was derived from an ascites sample from a 52-year-old female patient with iCCA (BTC-1). Immunofluorescent staining was performed for DAPI (blue) and pan-keratin (green). The organoid cultures then underwent WES and drug screening, and a ranked drug list was derived from the results (middle). This ranking includes information on targeted, endocrine, and chemotherapeutic agents and can be used by clinicians to guide treatment decisions (right). **B,** Clinical diagnoses of the cohort of patients with BTC. CCA-NOS, CCA, not otherwise specified; GBC, gallbladder carcinoma. **C,** Clinical stage of the patients with BTC. **D,** Clinical procedures used to collect the tumor organoids. **E,** Anatomic origin of the specimens. **F,** Treatment exposure history of patients with BTC prior to specimen collection. Any chemotherapeutic treatment, including adjuvant treatment, was counted as a previous treatment. **G,** Success rates of organoid establishment according to the clinical procedure used to obtain the specimen. **H,** Success rates of organoid establishment according to the anatomic site of origin. **I,** Overall success rates of organoid culture derivation and subsequent drug screening. The success rate was calculated for all specimens, including those that were found to have insufficient tumor content. The drug screening success rate was calculated from the number of established organoid cultures. [**A,** Created in BioRender. Kemp, C. (2026) https://BioRender.com/kv0dbe7.]

For this study, 46 live tissue samples were collected from 43 patients with BTC from 18 hospitals in the United States and shipped to derive tumor organoids for drug sensitivity testing utilizing the CLIA-certified PARIS assay (Fig. 1A; Supplementary Table S1). The median age at testing was 60.4 years (range, 36–82). Most specimens were derived from patients with iCCA (*n* = 31), and the remainder were from patients with eCCA (*n* = 5), gallbladder cancer (*n* = 6), CCA not otherwise specified (*n* = 3), and HCC-CCA (*n* = 1; Fig. 1B). Thirty-four patients had stage III or IV disease (79%), seven had stage I or II disease (16%), and the stages of five patients (11.6%) were unknown (Fig. 1C). Live tumor specimens were obtained from surgical resections (*n* = 21), core needle biopsies (*n* = 16), malignant ascites (*n* = 8), or punch biopsies (*n* = 1) from either primary tumors (*n* = 19) or distant metastases (*n* = 27; Fig. 1D and E). Fifteen specimens originated from patients who were treatment-naïve (32.6%), and 29 (63%) were from patients who had already progressed through chemotherapeutic or targeted treatments (range, 1–7 lines; Fig. 1F). The treatment history of two patients was unknown. The overall organoid establishment success rate was 58.7% (27/46). Failure to establish organoid cultures was most often due to insufficient tissue quantity or low tumor cell count and, to a lesser extent, to incorrectly frozen samples, insufficient washout periods between chemotherapy regimens and sample receipt, or microbiological contamination (Fig. 1G). Core needle biopsies were more prone to rejection due to lower tumor cell content (Fig. 1G). Although core biopsies generally had low tumor cell content and were prone to rejection, we observed better success rates with core samples specifically from liver or bile duct biopsies. Typically, the ascites samples proved to be more successful than core biopsies. The most successful samples were derived from liver or bile duct biopsies or ascites (Fig. 1H). For samples that passed initial quality control steps, organoids were established in 93.1% of cases (Fig. 1I). Of these, 96.3% (26/27) were screened with a customized drug panel (see “Materials and Methods”). For three patients, two specimens were obtained at different time points: BTC-8A and 8B, BTC-12A and BTC-12B, and BTC-15A and BTC-15B.

To confirm that the organoid cultures were representative of the patient’s tumor and to assess concordance between genetic biomarkers and drug sensitivity, WES was performed on PDTOs for which there was sufficient material after the completion of the PARIS test. Tumor origin was confirmed for 24 of the 26 organoids (92.3%; Supplementary Table S2). Twenty organoid cultures were confirmed by comparing WES-identified mutations to external genomic reports of the patient’s tumor. Four organoid cultures (BTC-8A, BTC-22, BTC-29, and BTC-39), which lacked external genomic data, were confirmed for tumor origin by the presence of multiple “tier 1” mutations, a designation referring to mutations observed in either the MC3 or GENIE databases and included in the OncoKB list of cancer genes. Due to limited tumor material, two organoid cultures (BTC-2 and BTC-9) had neither external genomics nor sufficient tissue for WES and could not be confirmed genomically. Overall, the spectrum and frequency of mutations in cancer genes found within our BTC cohort were similar to those reported by The Cancer Genome Atlas analysis of BTCs (Supplementary Table S3; ref. 18), indicating that this set of patient-derived organoids is representative of the genomic and clinical diversity of BTCs. Brightfield microscopy images of BTC 3D cultures are shown in Supplementary Fig. S1.

### The PARIS assay identified exceptional and patient-specific sensitivities to targeted agents in 92.3% of cases

**Figure 2.**
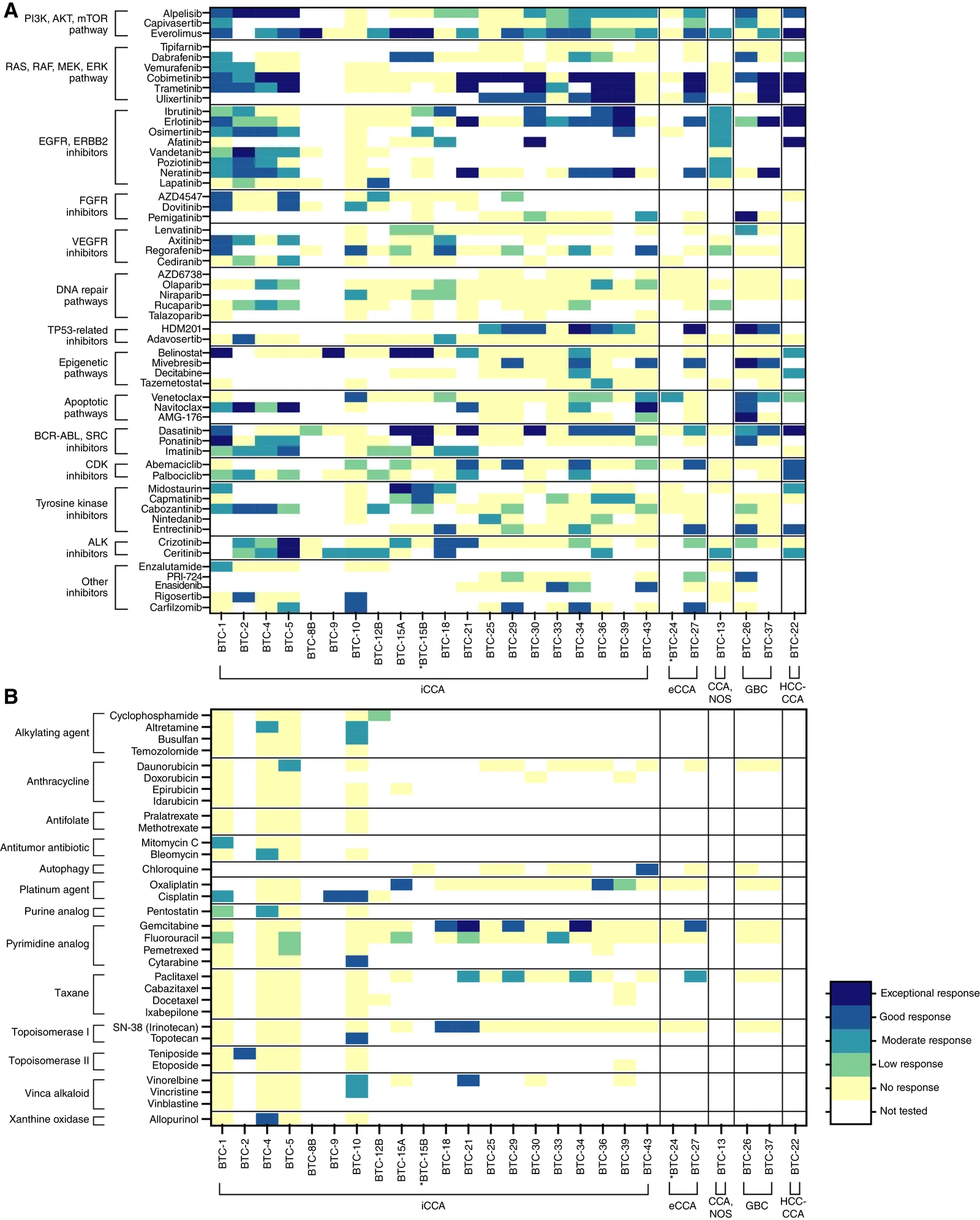
Pharmacotype of BTC organoids. **A,** Heatmap showing PARIS assay results for all targeted drugs tested in at least five organoid cultures. **B,** Heatmap showing PARIS assay results for all chemotherapeutic agents tested in at least one organoid culture. White rectangles indicate drugs that were not tested in that screen. Asterisks next to a patient number indicate that the drug screen failed some parameter of quality control but was still evaluable.

An average of 50 (range, 7–135) drugs at six doses were tested on 26 tumor organoid cultures, yielding more than 1,300 individual drug sensitivity assays for this patient cohort (Fig. 2; Supplementary Table S4). For each patient, a CLIA-certified report ranking drugs from the most to least effective was sent to the treating oncologists within a median of 34 days from receipt of the specimen. Remarkably, 92.30% (24/26) of organoid cultures exhibited a good-to-exceptional response (SPM 15-12) to at least one, and often more than one, FDA-approved targeted agent (Fig. 2A). Furthermore, 26 different targeted drugs showed good-to-exceptional activity in 25% or more of BTC 3D cultures tested (Table 1). These findings indicate a very high rate of actionability from *ex vivo* drug testing and reveal a broad menu of potentially effective drugs. Frequently active drugs included the ERK inhibitor ulixertinib (67% of PDTOs); the MEK inhibitors cobimetinib (58%) and trametinib (56%); the BCR-ABL and SRC inhibitor dasatinib (44%); multiple EGFR inhibitors including neratinib (42%), erlotinib (41%), osimertinib (33%), and afatinib (25%); the mTOR inhibitor everolimus (40%); and the BCL-2 inhibitor navitoclax (38%). Among the PI3K inhibitors, alpelisib, a PIK3CA-specific inhibitor, demonstrated activity in 27% of cases (Table 1). In contrast, the more broadly active PI3K pathway inhibitors, such as taselisib and buparlisib, were frequently active (∼80%; Table 1). However, the broad PI3K inhibitors, such as taselisib and buparlisib, have not advanced clinically due to limited efficacy and high toxicity (35). Additionally, several experimental drugs were commonly active, including the MDM2 inhibitor HDM-201 (64%) and the BET inhibitor mivebresib (60%).

**Table 1.** Frequently active drugs in BTC organoids.

Drug	Target	Proportion of good-to-exceptional responses
Taselisib	PI3K	83.3% (5/6)
Buparlisib	PI3K	80% (4/5)
NVP-BGT226	PI3K	80% (4/5)
Ulixertinib	ERK	66.7% (8/12)
HDM201	MDM2	63.6% (7/11)
Mivebresib	BET	60% (6/10)
BMS-754807	IGF-1R	60% (3/5)
Cobimetinib	MEK	58.3% (14/24)
Trametinib	MEK	55.6% (10/18)
Dasatinib	BCR-ABL, SRC	43.5% (10/23)
Neratinib	EGFR, ERBB2	42.1% (8/19)
Erlotinib	EGFR	40.9% (9/22)
Lucitanib	FGFR	40% (2/5)
Quizartinib	FLT3	40% (2/5)
Entinostat	HDAC	40% (2/5)
Everolimus	mTOR	40% (10/25)
Navitoclax	BCL2	38.5% (5/13)
Ibrutinib	BTK	35.7% (5/14)
Carfilzomib	Proteasome	33.3% (4/12)
Osimertinib	EGFR	33.3% (3/9)
Rigosertib	PLK1	33.3% (2/6)
Abemaciclib	CDK	30% (6/20)
Alpelisib	PI3K	27.3% (6/22)
Entrectinib	ROS1, TRK	25% (4/16)
Afatinib	EGFR	25% (2/8)
Dovitinib	FGFR	25% (2/8)

NOTE: Drugs with good-to-exceptional responses in ≥25% of organoid cultures are listed. Drugs that were tested in less than five cultures were excluded.

In addition to these shared dependencies, each organoid culture exhibited a unique pharmacotype across the entire panel of drugs, reflecting the genetic and phenotypic heterogeneity that exists between different patients with BTC (Fig. 2). Also, although consistent responses were typically observed within a given drug class, preferences for specific EGFR, FGFR, or MEK inhibitors were seen, which likely reflects different mechanisms of action of these targeted inhibitors.

In contrast to frequent sensitivities to targeted agents, BTC organoids were largely resistant to the chemotherapeutic agents tested (Fig. 2B). Of 232 individual drug sensitivity assays comprising 32 different chemotherapeutic drugs, only 17 assays (7.3%) showed a good-to-exceptional response. More frequently active chemotherapy drugs included cisplatin and gemcitabine, both of which are FDA-approved as first-line BTC treatments. Overall, these findings are consistent with the generally poor clinical response of BTCs to chemotherapies (16).

### Patient-derived 3D tumor cultures enable functional assessment of drug resistance in the retrospective setting

Previous studies addressing drug resistance have primarily relied on the use of model systems that are genetically unrelated to any given patient with cancer or on descriptive analysis of molecular or genetic alterations in recurrent, drug-resistant tumors (36). However, these approaches typically cannot establish causality as they lack functional validation and, moreover, rarely point to effective strategies to overcome drug resistance. For example, the extent to which clinical drug resistance is attributed to tumor cell-autonomous resistance mechanisms or to the tumor microenvironment or other host factors is poorly understood. To determine if PDTOs can be used to functionally model drug resistance, we first compared the drug sensitivity landscape between organoids derived from treatment-naïve and previously treated patients. Organoids derived from treatment-naïve patients showed exceptional responses to an average of 3.8 drugs, whereas organoids derived from previously treated patients were responsive to an average of 4.5 drugs, mostly targeted agents. These findings indicate that tumor cells derived from previously treated patients remain sensitive to the inhibition of a variety of oncogenic signaling pathways and that the development of resistance to standard-of-care treatments does not result in a universally drug-resistant phenotype.

Next, we assessed whether tumor-derived organoids phenocopy the patients’ clinical resistance to specific therapies. For eight patients, we compared organoid sensitivities to chemotherapies the patients had previously taken and on which the patients experienced disease progression prior to tissue collection for organoid derivation (Table 2). Three patients had progressed on capecitabine, a prodrug of fluorouracil. Organoids derived from all three patients (BTC-15A, BTC-21, and BTC-36) had low or no response to fluorouracil. Three patients (BTC-12B, BTC-30, and BTC-39) were previously treated with FOLFOX, folinic acid, fluorouracil and Irinotecan (FOLFIRI), or folinic acid, fluorouracil, irinotecan, and oxaliplatin (FOLFIRINOX). Organoid cultures derived from these three patients showed low or no response to all drugs from these regimens (fluorouracil, irinotecan, and oxaliplatin) tested as single agents. Six patients received and progressed on gemcitabine with or without cisplatin. Organoids derived from four of these patients, BTC-1, BTC-36, BTC-37, and BTC-39, showed low or no response to one or both drugs. BTC-21 organoids had an exceptional response to gemcitabine, a drug the patient progressed on 3 years prior to the PARIS test, and no sensitivity to the platinum drug, oxaliplatin. BTC-15A organoids were insensitive to gemcitabine but showed a good response to oxaliplatin. The latter two cases may suggest that clinical resistance to combination chemotherapies could be due to resistance to only one drug, something we have observed for other cancer types. In summary, 21/24 (87.5%) drugs taken previously by patients with BTC showed no (70.8%) or low (16.7%) activity in subsequently tested PDTOs, indicating that clinical drug resistance is phenocopied by resistance to the same drugs in the tumor cells themselves. These findings indicate that PDTOs can be used to functionally assess patient-specific drug resistance while simultaneously identifying additional, potentially effective drugs.

**Table 2.** Retrospective analysis of preassay therapies and PDTOs drug sensitivity.

PDTC	Preassay treatments with clinical progression	*Ex vivo* drug response	
		Drug tested	SPM score
BTC-1	Gemcitabine + cisplatin	Gemcitabine	4
		Cisplatin	10
BTC-12B	Gemcitabine + paclitaxel	Gemcitabine	5
	Radiation with capecitabine	Fluorouracil	1
	FOLFOX and FOLFIRINOX	Cisplatin	6
		Fluorouracil	1
BTC-15A	Capecitabine	Fluorouracil	9
	Gemcitabine + cisplatin	Gemcitabine	5
		Oxaliplatin	13
	Olaparib	Olaparib	1
BTC-21	Gemcitabine + cisplatin	Gemcitabine	14
		Oxaliplatin	5
	Capecitabine	Fluorouracil	9
BTC-30	FOLFIRINOX	Fluorouracil	2
		Oxaliplatin	1
		SN-38 (irinotecan)	2
BTC-36	Capecitabine	Fluorouracil	3
	Gemcitabine	Gemcitabine	4
	Irinotecan	SN-38 (irinotecan)	2
BTC-37	Gemcitabine + cisplatin	Gemcitabine	4
		Oxaliplatin	1
BTC-39	Gemcitabine + cisplatin and gemcitabine + carboplatin	Gemcitabine	4
		Oxaliplatin	9
	FOLFIRI	SN-38 (irinotecan)	3

NOTE: Eight patients who progressed on previous treatments provided specimens in which organoids were subsequently tested with the same or surrogate agents. All *ex vivo* drug responses were to single agents and not the combinations taken by some patients.

### Biomarker/drug sensitivity associations

**Figure 3.**
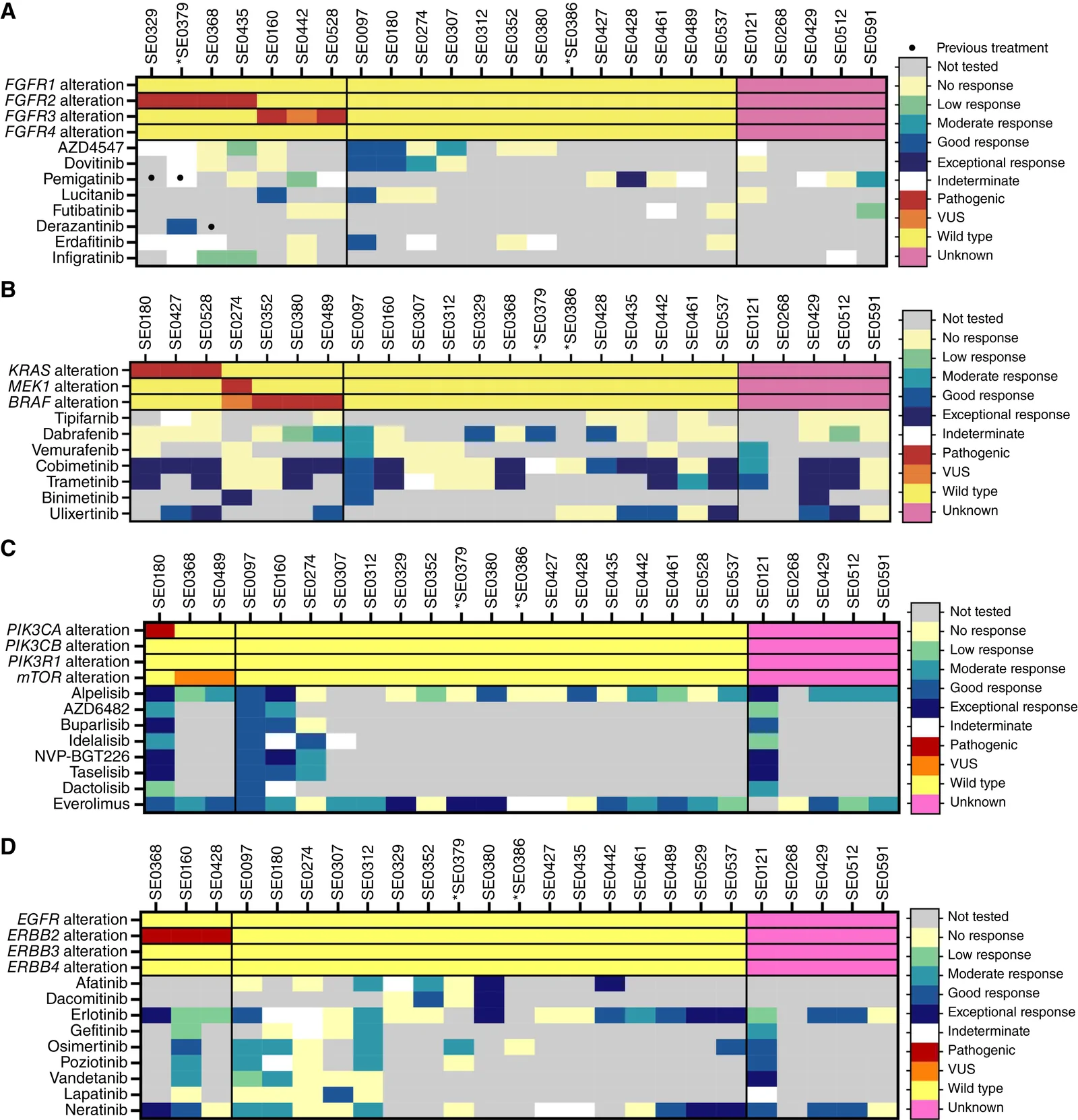
Concordance of *ex vivo* drug response and genetic biomarkers. **A,** Heatmap of responses to the FDA-approved FGFR inhibitors futibatinib, infigratinib, and pemigatinib, as well as other FGFR inhibitors derazantinib, AZD4547, dovitinib, lucitanib, and erdafitinib. Dasatinib was included because, as shown here, sensitivity has been detected in several tumors with FGFR alterations that are clinically resistant to FGFR inhibitors. Alterations in *FGFR1*, *FGFR2*, and *FGFR3* are annotated. **B,** Heatmap of responses to the HRAS inhibitor tipifarnib; the BRAF inhibitors vemurafenib and dabrafenib; the MEK inhibitors cobimetinib, trametinib, and binimetinib; and the ERK inhibitor ulixertinib. Alterations in *KRAS*, *NRAS*, *BRAF*, and *MEK1* are annotated. **C,** Heatmap of responses to the PI3K inhibitors alpelisib, AZD6482, buparlisib, idelalisib, NVP-BGT226, taselisib, the PI3K/mTOR inhibitor dactolisib, and the mTOR inhibitor everolimus. Alterations in *PIK3CA* and *PIK3CB* are annotated. **D,** Heatmap of responses to the EGFR inhibitors afatinib, dacomitinib, erlotinib, gefitinib, osimertinib, poziotinib, and vandetanib and the ERBB2 inhibitors lapatinib and neratinib. Alterations in *EGFR*, *ERBB2*, *ERBB3*, and *ERBB4* are annotated. Asterisks next to a patient number signify that the screen failed a measure of quality control but was still evaluable.

We next evaluated the concordance between *ex vivo* drug sensitivity and corresponding genomic biomarkers, including alterations in FGFR, KRAS/NRAS, BRAF, MEK, PIK3CA, and EGFR/ERBB2. Ivodisenib, which targets mutant IDH1, could not be evaluated in our cell viability–based assay as it induces cellular differentiation as opposed to cell death (37). The FGFR inhibitors futibatinib, pemigatinib, and infigratinib are approved for BTC with FGFR2 fusions or rearrangements. We tested these and other FGFR inhibitors that are not FDA-approved, including derazantinib, AZD4547, erdafitinib, dovitinib, and lucitanib. Four tumor samples had reported FGFR fusions: BTC-15, BTC-21, and BTC-29 (Fig. 3A). Patient BTC-15 was diagnosed with an iCCA carrying an *FGFR2-BICC1* fusion and failed to respond to several lines of treatment including pemigatinib. One and a half years after diagnosis, tumor organoids were derived from the patient’s ascites and subjected to the PARIS assay. The results indicated no response to several FGFR inhibitors consistent with the patient’s recent progression on pemigatinib. In contrast, several targeted therapies that the patient had not been exposed to demonstrated exceptional responses, including dasatinib (Fig. 2A), which is discussed further below.

**Figure 4.**
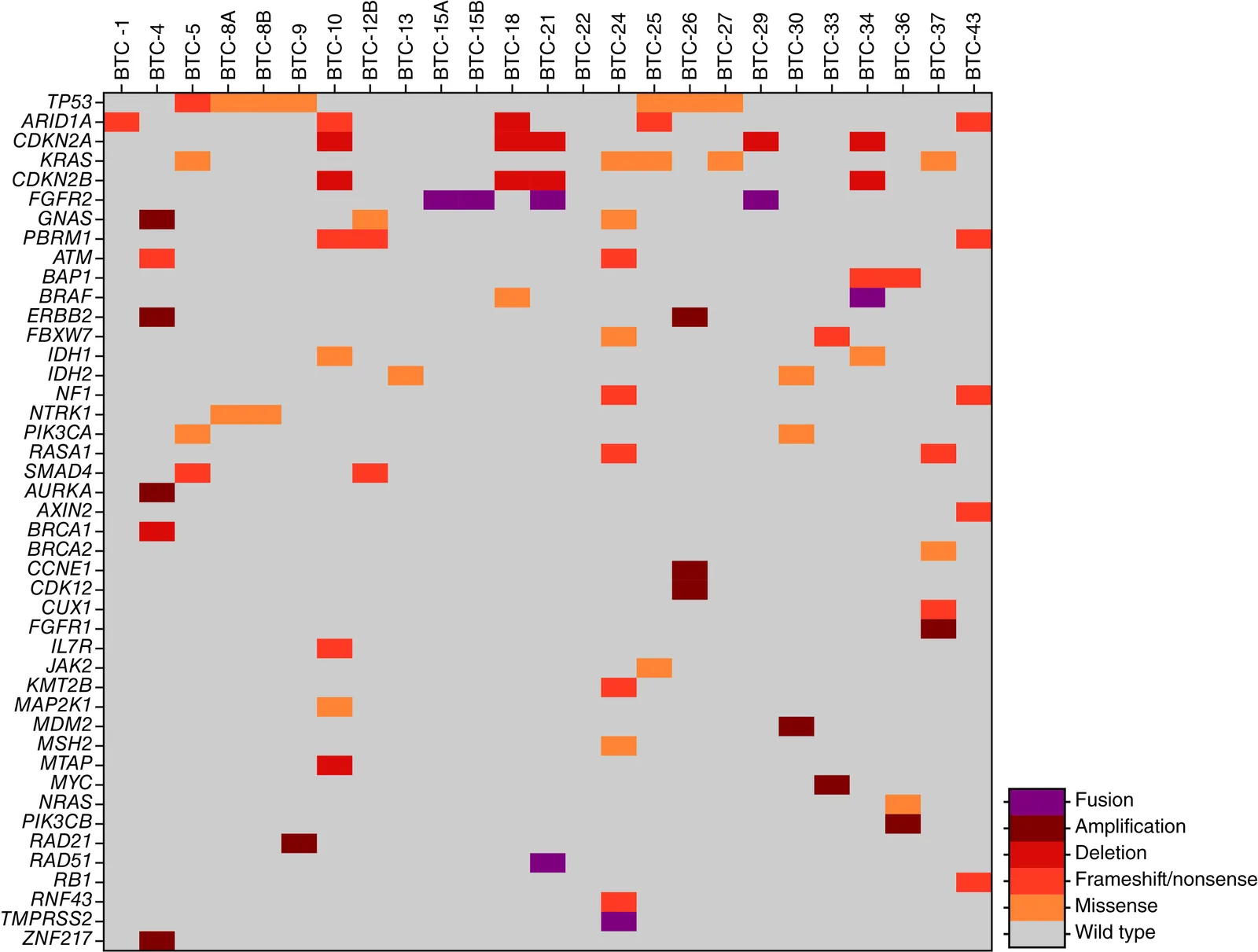
OncoPrint for the BTC cohort. All structural rearrangements, pathogenic mutations, copy gains, and copy losses of genes taken from clinical histories and third-party NGS sequencing for the BTC cohort are noted. VUSs are not included.

Patient BTC-21 was diagnosed with an iCCA harboring an FGFR2-KCNH7 fusion and progressed after 9 months of treatment with derazantinib. At the time of progression, organoids were established for the PARIS assay, and results showed no response to several FGFR inhibitors: AZD4547, dovitinib, and erdafitinib, and a low response to infigratinib (Fig. 4A). Derazantinib was not tested. As with BTC-15A, BTC21 also had an exceptional response to dasatinib and other targeted agents (Fig. 2A). The exceptional sensitivity to dasatinib in organoids from these two patients with *FGFR* fusions who had progressed on FGFR-directed therapies was also observed in two additional PDTOs: one with an *FGFR3* variant of unknown significance (VUS; BTC-30) and one with an *FGFR1* amplification (BTC-37; Fig. 3A). Overall, 43.5% (10/23) of BTC organoids showed a good-to-exceptional response to dasatinib (Table 1).

A possible false-negative response was observed with BTC-29, a treatment-naïve patient harboring an FGFR2-SORBS1 fusion. Unfortunately, there was insufficient sample to evaluate if the organoid culture carried the FGFR2-SORBS1 fusion. Organoids derived from this patient showed low sensitivity to AZD4547 and infigratinib. Additionally, a partial ∼20% response was observed with pemigatinib, which did not meet the criteria for a top drug response (Fig. 3A). Soon after the PARIS assay, the patient progressed through capecitabine and subsequently through combination gemcitabine + cisplatin therapy. The organoids, derived prior to these treatments, did not show a response to fluorouracil and oxaliplatin (cisplatin was not tested) and had a moderate response to gemcitabine. One year after the PARIS assay, the patient received pemigatinib with a significant durable response.

We next asked if PDTOs could be used to inform the association between biomarkers and drugs that are FDA-approved for cancers other than BTC. Five patients with BTC harbored pathogenic *RAS* mutations, including BTC-5 (*KRAS* Q61H), BTC-25 (*KRAS* G12D), BTC-27 (*KRAS* Q61H), BTC-36 (*NRAS* Q61R), and BTC-37 (*KRAS* G12C). Mutant KRAS-specific inhibitors were not available at the time of this study; thus, MEK and ERK inhibitors were used as indicators of mutant KRAS-induced MAPK signaling. Organoid cultures from all five patients showed exceptional sensitivities to the MEK inhibitors cobimetinib, trametinib, and binimetinib and the ERK inhibitor ulixertinib (Fig. 3B).

Patient BTC-5 carried a pathogenic *PIK3CA* E545K mutation, and corresponding organoids showed exceptional/good responses to the four PI3K inhibitors tested, as well as the mTOR inhibitor everolimus (Fig. 3C). BTC-30 harbored a pathogenic *PIK3CA* C420R mutation, and tumor-derived organoids showed moderate sensitivity to both alpelisib and everolimus.

Two patients harbored *ERBB2* gene amplifications. BTC-4, an iCCA, was sensitive to six drugs that target the EGFR/ERBB2 (HER2) proteins, with good responses to the irreversible inhibitors neratinib and osimertinib (Fig. 3D). BTC-26, a gallbladder cancer, showed a moderate response to erlotinib and no response to neratinib (Fig. 3D).

Finally, many BTC organoid cultures showed high sensitivity to targeted agents in which alterations in the corresponding target gene were absent or the status was unknown, indicating the need for functional testing. For example, 11 out of 16 BTC organoids without known *KRAS*, *NRAS*, *BRAF*, or *MEK1* alterations showed good-to-exceptional responses to MAPK-targeted drugs (Fig. 3B), suggesting alternate mechanisms of MAPK pathway dependency. Additionally, 11 out of 20 BTC organoid cultures showed good-to-exceptional sensitivity to PI3K or mTOR inhibitors without apparent alterations of these genes (Fig. 3C). As we focused on the gene-specific analysis and known single-nucleotide variants and did not assess larger structural arrangements, such as chromosomal arm gain or loss, we were unable to determine whether these genomic features contributed to the observed drug response. Collectively, these findings illustrate how PDTOs can be used to evaluate associations between drug sensitivities and genetic biomarkers, including VUSs. Importantly, because PDTOs are genetically faithful models of any given patient’s tumor, these drug/biomarker associations are carried out in the appropriate genetic context.

### Use of the PARIS assay to guide therapy

For five iCCA stage IV patients, all of whom had progressed through multiple lines of treatment, the treating oncologists decided to use the results of the PARIS assay to guide therapy selection (Table 3). For all five patients, the PARIS assay identified at least two or more drugs with good-to-exceptional activity, indicating potential actionability. Although their oncologists were ultimately able to obtain compassionate use of these off-label drugs from pharmaceutical companies, the process was prolonged, causing significant treatment delays for these terminally ill patients. Three patients who received PARIS-guided treatments experienced drug toxicities and had to discontinue treatment. Here, we describe two patients who were able to remain on PARIS-guided treatment for more than 4 weeks.

**Table 3.** Summary of patients who received treatments guided by PARIS assay results.

Patient				Screen		*Ex vivo* drug response		Treatment guided by PARIS test
Patient	Diagnosis and stage	Previous treatment lines	Genomics	Drugs tested	Turnaround time	Top scoring drugs	SPM score	
BTC-8B	Stage IV iCCA, metastases to the liver and lungs	3	Unknown	14	16 days	**Everolimus**	**15**	​
						AZD5363	12	**Everolimus**
						Crizotinib	9	​
						MK2206	5	Discontinued due to side effects at 10 weeks
						Momelotinib	4	
						Taselisib	2	
BTC-10	Stage IV iCCA, metastases to the skin, liver, soft tissue, and bones	4	*ARID1A* (L1712fs*2) *CDKN2A* loss *CDKN2B* loss *IDH1* (R132S) *MAP2K4* (Q56P) *MTAP* (exon 5–8 loss) *PBRM1* (Q1273*)	135	9 days	**Binimetinib**	**14**	​
						Carfilzomib	13	​
						Cisplatin	13	​
						Oprozomib	13	​
						Ribociclib	13	**Trametinib and hydroxychloroquine**
						Cytarabine	12	
						Ravoxertinib	12	​
						Idelalisib	12	Discontinued due to drug toxicity after 2 weeks
						Regorafenib	12	
						Topotecan	12	​
						Venetoclax	12	​
						Apitolisib	11	​
						Niraparib	11	​
						MK2206	10	​
BTC-15A	Stage IV iCCA, metastases to the liver and peritoneum	5	*FGFR2-BICC1* fusion *FGFR2* amplification *TP53* (673-1G>A)	48	45 days	Belinostat	15	​
						**Dasatinib**	**15**	**Dasatinib and bevacizumab**
						Crenolanib	14	​
						Entinostat	14	Improved symptoms and stable disease, discontinued due to disease progression after 11 weeks
						Everolimus	14	
						Dabrafenib	13	
						Midostaurin	13	Overall survival 8.5 months
						Oxaliplatin	13	​
BTC-36	Stage IV iCCA, metastases to the lungs, liver, and peritoneal cavity	2	*NRAS* (Q61R) *BAP1* (P153fs*34) *PIK3CB* amplification	46	48 days	Cobimetinib	15	​
						**Ulixertinib**	**15**	​
						Trametinib	14	​
						Erlotinib	13	**Ulixertinib**
						HDM201	13	​
						Ibrutinib	12	Discontinued due to drug toxicity after 9 days
						Neratinib	12	
						Dasatinib	12	​
						Lorlatinib	11	​
						Alpelisib	10	​
BTC-39	Stage IV iCCA, metastasis to liver	2	*IDH1* R132C	45	63 days	Ulixertinib	15	​
						**Neratinib**	**15**	**Neratinib**
						Cobimetinib	15	Discontinued due to drug toxicity after 5 weeks
						Erlotinib	14	
						**Trametinib**	14	​
						Osimertinib	13	**Trametinib**
						Alpelisib	11	Patient passed away after 5 weeks
						HDM-201	10	
						Capmatinib	9	​

NOTE: Five patients were able to obtain off-label use of drugs informed by *ex vivo* drug testing. The diagnosis, disease stage, previous treatment lines, known genomics, number of drugs tested *ex vivo*, time from receipt of specimen to delivery of report, top scoring drugs, SPM scores, and subsequent treatments are shown. All organoid cultures showed good-to-exceptional responses to multiple drugs despite being derived from patients who had progressed through multiple treatment lines. BTC-10 was unable to obtain the top scoring drug, binimetinib, and obtained another MEK inhibitor, trametinib, which had a low SPM score of 7. Two patients had at least two months of symptom reduction before disease progression. Three patients were unable to tolerate the toxicity of the chosen drug at their stage of disease and had to discontinue treatment. Bold text represents the drug patient has taken, only in second case it's same class of drugs not the exact drug. The numbers represent the SPM score of the drug patient received, for second case Binimetinib should be considered.

The first case, a 64-year-old female (BTC-8B), had progressed on gemcitabine, cisplatin, and FOLFOX treatments and lacked actionable genomic alterations as assessed by a third-party liquid biopsy. The PARIS assay indicated two drugs: everolimus and taselisib, with good-to-exceptional activity, and the oncologist selected everolimus for further treatment. Clinic notes at 5 weeks after treatment reported that the patient experienced a significantly decreased symptom burden. In particular, the rate of ascites accumulation decreased from 1 L per day to only requiring paracentesis every 2 weeks. The patient’s pain also became more manageable, and the patient was able to discontinue all pain medication. This improvement was reported through 8 weeks of treatment. At 10 weeks, the patient stopped everolimus treatment due to severe fatigue and entered hospice care. Overall, the patient survived 3 months from the start of PARIS-guided treatment.

The second case was a 43-year-old female (BTC-15A) with an *FGFR2-BICC1* fusion who did not tolerate gemcitabine and cisplatin, had undergone multiple surgeries, and had progressed on pressurized intraperitoneal aerosolized chemotherapy (PIPAC) with doxorubicin and cisplatin, systemic olaparib, and the FGFR inhibitor pemigatinib. At 1.5 years from diagnosis, PDTO drug testing derived from malignant ascites confirmed resistance to FGFR inhibitors but indicated exceptional responses to several targeted agents, including dasatinib. The oncologist selected dasatinib, which was given in combination with bevacizumab. Following this regimen, the patient experienced symptom improvements and stable disease for 9 weeks. Overall, the patient survived 8.5 months from the start date of PARIS-informed treatment. These case examples indicate that *ex vivo* drug testing provides actionable drug choices even for patients with late-stage disease and can be used to initiate assay-guided care with clinical benefit.

## Discussion

The PARIS test represents a functional precision oncology approach that complements genomic profiling by directly assessing the sensitivity of live patient-derived tumor cells to therapeutic agents *ex vivo*. In CCA, in which molecular heterogeneity, lineage diversity, and acquired resistance frequently limit the predictive value of genomic alterations alone, functional testing may capture clinically relevant drug–tumor interactions that are not readily inferred from sequencing data. Although genomic profiling can identify actionable alterations in a subset of patients (e.g., FGFR2 fusions, IDH1 mutations, HER2 alterations), many patients lack actionable targets or demonstrate discordant clinical responses despite the presence of predicted biomarkers. Unlike sequencing-based approaches that infer treatment response from molecular features, the PARIS Test evaluates the integrated biological behavior of the tumor, incorporating the effects of tumor genetics, epigenetic state, resistance mechanisms, and cellular adaptations within the patient’s current disease context. By testing a personalized panel of oncology therapies and ranking drug sensitivity profiles, the assay may help prioritize treatment options with a greater likelihood of clinical activity while reducing exposure to potentially ineffective therapies.

A critical parameter for the real-world deployment of *ex vivo* assays in advanced gastrointestinal oncology is procedural feasibility and clinical turnaround time. Data from the PARIS platform demonstrate that actionable reports ranking drug sensitivity profiles are delivered within an average window of 3 to 4 weeks. This time frame aligns with standard-of-care restaging intervals, making it highly viable for patients transitioning to second- or third-line regimens in which standardized guidelines are lacking. The platform exhibits wide-ranging versatility across sample acquisition modalities, requiring four core needle biopsies, a surgical resection measuring 1 to 1.5 cm, or ∼250 mL of malignant body fluids (such as ascites or pleural effusions). This accommodates the complex anatomic constraints often encountered during biliary tract interventions.

BTCs remain difficult to treat due to poor responses to both first- and second-line chemotherapies. Next-generation sequencing has revealed a complex genetic landscape in BTCs, providing a foundation for biomarker-guided treatments. However, except for agents targeting *FGFR*, *IDH1*, and *HER2* alterations and infrequently MSI and NTRK alterations, biomarker-associated therapies remain limited. This multicenter study demonstrates the feasibility and clinical utility of organoid-based drug testing to assess biomarkers, model drug resistance, and identify potential therapies for patients with BTC. Samples were provided by patients from 18 different hospitals, and organoids were successfully derived and screened from multiple BTC subtypes and tumor stages, including at diagnosis, when surgery is often the first intervention, and from late-stage disease when paracentesis or liver biopsies may be performed. Samples were derived from both treatment-naïve and heavily pretreated patients from multiple anatomic sites. Genomic alterations detected in PDTOs mirrored those reported for other BTC cases, indicating that this set of organoids was largely representative of the clinical and genetic diversity of patients with BTC. When patient-derived tumor samples contained sufficient live tumor cells and passed initial quality control steps, the assay’s success rate was 93.2%, with a median turnaround time from sample arrival to CLIA report of 34 days. This success rate and time frame are comparable with other precision medicine tests and compatible with informing treatment options. However, given the fast progression of refractory BTCs, performing the PARIS test earlier, especially if surgery is indicated, would be ideal. Although most tumor organoids were derived from patients with late-stage disease who had undergone prior treatments, nearly 100% exhibited significant vulnerabilities to at least one or more targeted agents. Overall, the cohort showed sensitivities to a broad range of oncogenic pathway inhibitors, including MEK/ERK, EGFR/HER2, and MDM2 inhibitors, as well as broad-spectrum TKIs such as dasatinib, for which sensitivity was observed in 5 out of 7 PDTOs with FGFR translocations (Fig. 4). This observation, especially from after FGFRi therapy, could indicate that the activation of SRC family kinases may be a mechanism of drug resistance. Indeed, dasatinib has been previously observed to sensitize urothelial carcinoma to the FGFR inhibitor infigratinib (38) and to overcome resistance to ibrutinib in lymphomas (39) and to IDH1 inhibitors in CCA (40). Although ERK inhibitors are still experimental and limited by severe side effects, MEK inhibitors could be used in combination strategies. For example, sensitivity to both MEK and EGFR inhibitors co-occurred in 10 out of 15 PDTOs, and these drugs can be used in combination, an approach that is being adopted to treat colorectal and other cancers (41).

Targeted agents showed good-to-exceptional activity in PDTOs, which harbored pathogenic mutations in corresponding gene targets such as RAS, FGFR, PI3K, and EGFR/ERBB2. Targeted agents were also effective in samples that harbored VUSs and samples that did not harbor the targeted pathogenic activating mutations, suggesting that other mechanisms beyond single-gene mutations can generate pathway dependencies. Future research will be needed to understand the observed functional activation of specific pathways in the absence of pathognomonic alterations.

However, because patient-derived tumor cells share the same complex genetic landscape and treatment history with any given patient’s tumor, and because each tumor’s genetic makeup can modify drug sensitivity, the use of PDTOs may augment genomic-based drug sensitivity predictions. For example, a pancreatic cancer case with *ERBB2* amplification coexisting with a *KRAS* mutation received ERBB2-directed therapy based on functional testing results, leading to a complete and durable response to treatment (42). In another example, a terminally ill patient with ovarian cancer and no actionable genetic biomarkers had a dramatic and sustained clinical response to ibrutinib, which was the top-scoring drug in her organoid-based PARIS assay (23).

Oncologists used PARIS assay results to guide therapy for five patients with late-stage BTC. Two of these patients were able to remain on treatment for 8 and 10 weeks, experienced a period of symptom improvement, and lived 3 and 8 months, respectively. The compromised health, associated drug toxicity, and delay in obtaining off-label drugs made it difficult to assess clinical benefit in three additional patient cases. In the future, using functional precision medicine assays earlier in the patient’s clinical journey and ready access to off-label drugs will facilitate the assessment of clinical benefit. This first-generation BTC organoid pharmacotype provides a functional map of patient-specific vulnerabilities and therapeutic opportunities. Personalized drug combinations targeting both genomic and functional biomarkers could be beneficial for patients, especially those who fail standard-of-care treatments.

These findings support positioning the *ex vivo* drug testing approach as a complementary, synergistic component of a multiomic precision oncology framework. Compared with other assays in functional precision medicine, the PARIS test has the capacity to measure a highly personalized broad panel of drugs (up to 47 drugs or larger), based on the unique genetics, clinical history of the patient, oncologists’ recommendations, and drug availability. Additionally, because the assay is based on unique software and accurate acoustic robotics, it allows for a highly controlled, error-free system that is scalable to incorporate more drugs or drug combinations. Finally, the WES verification of organoids just prior to the PARIS assay provides additional information to oncologists, beyond the limited gene panels utilized in precision medicine. It will also be instrumental for research to identify novel and potentially complex biomarkers of drug response. A limitation of PDTO-based drug sensitivity testing is that it evaluates tumor behavior outside the tumor microenvironment. Nonetheless, several case studies suggest that integrating PARIS functional drug response with or without genomic profiling indications can inform effective treatment selection and yield durable clinical responses in refractory malignancies (23, 42).

Future prospective studies in BTCs should adopt a coclinical design integrating real-time functional sensitivity profiles with longitudinal, serial molecular profiling to determine whether this combined approach can improve treatment response, reduce exposure to ineffective therapies, and ultimately enhance clinical outcomes.

## Supplementary Material

Figure S1 Brightfield images of BTC organoid cultures

Table S1 Clinical characteristics of the BTC cohort

Table S2 Tumor organoid confirmation by comparison to third party genomics of the primary tumor

Table S3 Supplementary Table 3. Cholangiocarcinoma genomic landscape from The Cancer Genome Atlas (TCGA) and comparison to this BTC cohort.

Table S4 Supplementary Table S4. Drugs tested for each tumor organoid culture.

## Data Availability

Deidentified data used in the research were collected in a real-world healthcare setting and are subject to controlled access for privacy and proprietary reasons. Deidentified WES data may be made available from the corresponding author upon reasonable request and subject to institutional approval, data use agreements, and applicable ethical and legal requirements.
