# Supplementary material for: Ex Vivo Tumor-Derived Organoid Pharmacotyping Identifies Personalized Therapeutic Options for Patients with Biliary Tract Cancer
Source: Cancer Res Commun. 2026 Aug 31;6(8):2026–38. doi: 10.1158/2767-9764.CRC-25-0792 (PMC13528702; doi:10.1158/2767-9764.CRC-25-0792)

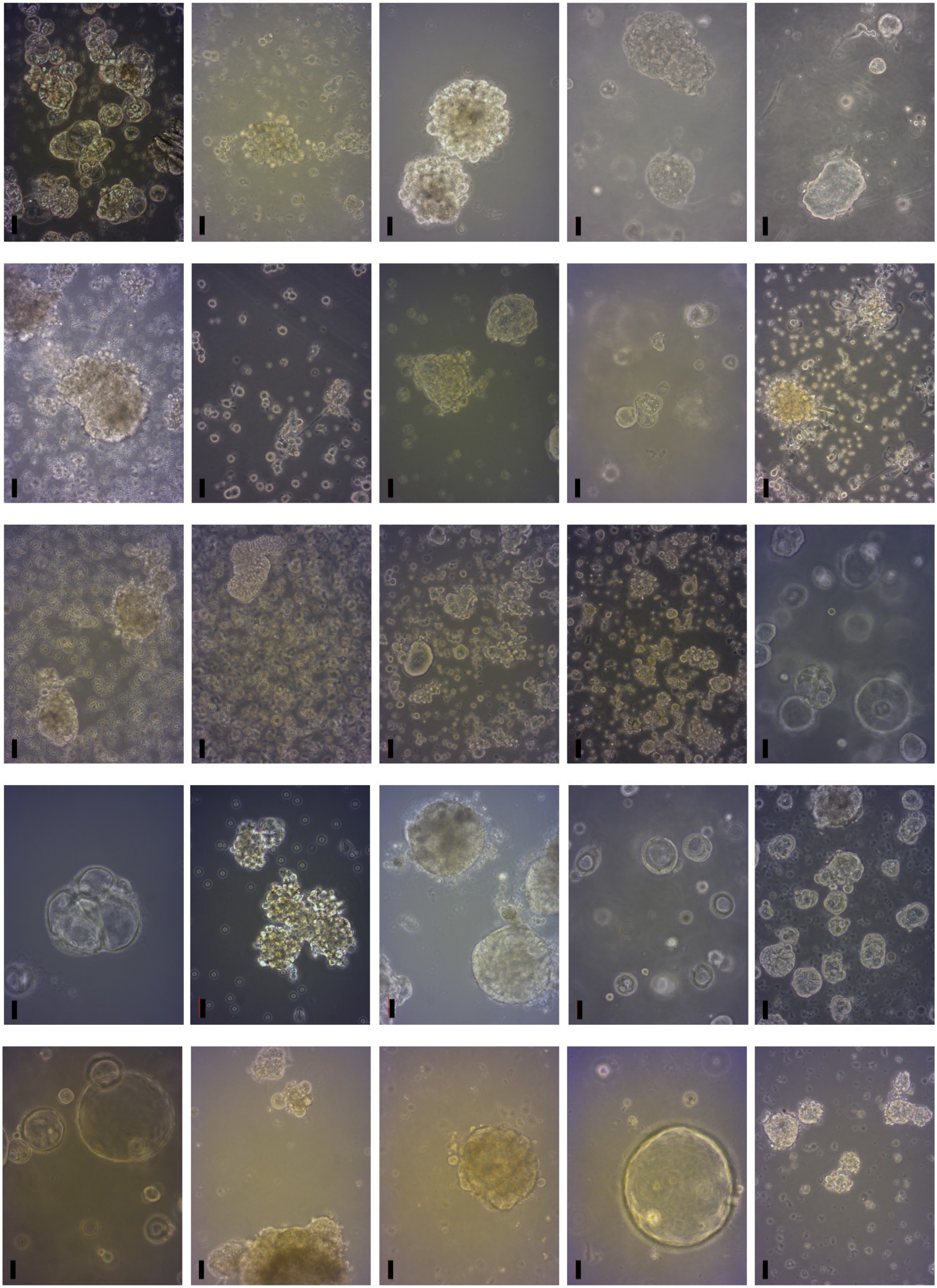

Supplement: Supplementary file 1 [file crc-25-0792_figure_s1_suppsf1.docx]
