# Supplementary material for: Ex Vivo Tumor-Derived Organoid Pharmacotyping Identifies Personalized Therapeutic Options for Patients with Biliary Tract Cancer
Source: Cancer Res Commun. 2026 Aug 31;6(8):2026–38. doi: 10.1158/2767-9764.CRC-25-0792 (PMC13528702; doi:10.1158/2767-9764.CRC-25-0792)
Supplement: Supplementary file 2 [file crc-25-0792_table_s1_suppst1.docx]

| **Supplemental Table 1. Summary of clinical characteristics of BTC cohort** |  |  |
| --- | --- | --- |
| **Cohort characteristics** | **Number** | **Percentage** |
| Total number of patients | 43 | - |
| Total number of samples | 46 | - |
| Total number of screens | 49 | - |
| **Age** | | |
| Median age at diagnosis | 52.2 | - |
| Median age at screening | 60.4 | - |
| **Gender** | | |
| Male | 25 | 58.1 |
| Female | 18 | 41.9 |
| **Clinical diagnosis** | | |
| Intrahepatic cholangiocarcinoma | 31 | 67.4 |
| Extrahepatic cholangiocarcinoma | 5 | 10.9 |
| Gallbladder adenocarcinoma | 6 | 13.0 |
| Cholangiocarcinoma, NOS | 3 | 6.5 |
| Combined hepatocellular cholangiocarcinoma | 1 | 2.2 |
| **Stage** | | |
| Stage I | 2 | 4.4 |
| Stage II | 5 | 10.9 |
| Stage III | 6 | 13.0 |
| Stage IV | 28 | 60.9 |
| Unknown | 5 | 10.9 |
| **Specimen source** | | |
| Primary tumor | 19 | 41.3 |
| Distant metastasis | 27 | 58.7 |
| **Specimen anatomical site** | | |
| Ascites | 8 | 17.4 |
| Bile duct | 18 | 39.1 |
| Gallbladder | 2 | 4.4 |
| Liver | 9 | 19.6 |
| Lymph node | 1 | 2.2 |
| Peritoneum | 3 | 6.5 |
| Pancreas | 1 | 2.2 |
| Skin | 1 | 2.2 |
| Soft tissue | 3 | 6.5 |
| **Treatment exposure** | | |
| One or more treatment lines | 29 | 63.0 |
| Naïve of treatment | 15 | 32.6 |
| Unknown | 2 | 4.3 |
