# Supplementary material for: Ex Vivo Tumor-Derived Organoid Pharmacotyping Identifies Personalized Therapeutic Options for Patients with Biliary Tract Cancer
Source: Cancer Res Commun. 2026 Aug 31;6(8):2026–38. doi: 10.1158/2767-9764.CRC-25-0792 (PMC13528702; doi:10.1158/2767-9764.CRC-25-0792)
Supplement: Supplementary file 3 [file crc-25-0792_table_s2_suppst2.docx]

| **Supplementary Table S2. Title: Genomic Confirmation of patient derived organoids via WES** | | | |
| --- | --- | --- | --- |
|  |  |  |  |
| **BTC Sample No.** | **3rd Party Mutations Confirmed** | **Tier 1 Mutations Detected** | **FGFR Translocations reported by 3rd Party NGS** |
|  |  |  |  |
| BTC 1 | 52.60% | Confirmed by 3rd Party Concordance | Not Reported |
| BTC 2 | Not enough material for DNA |  | Not Tested |
| BTC 4 | 100% | Confirmed by 3rd Party Concordance | Not Reported |
| BTC 5 | 90% | Confirmed by 3rd Party Concordance | Not Reported |
| BTC 8A | 50% | Confirmed by 3rd Party Concordance | Not Reported |
| BTC 9 | Not enough material for DNA |  | Not Tested |
| BTC 10 | 65% | Confirmed by 3rd Party Concordance | Not Reported |
| BTC 12B | 28.60% | Confirmed by 3rd Party Concordance | Not Reported |
| BTC 13 | 70% | Confirmed by 3rd Party Concordance | Not Reported |
| BTC 15A | 71.40% | Confirmed by 3rd Party Concordance | *FGFR2-BICC1* |
| BTC 18 | 100% | Confirmed by 3rd Party Concordance | Not Reported |
| BTC 21 | 77.80% | Confirmed by 3rd Party Concordance | *FGFR2-KCNH7* |
| BTC 15B | 71.40% | Confirmed by 3rd Party Concordance | *FGFR2-BICC1* |
| BTC 22 | confirmed by TIER1 mutations | Confirmed by Tier 1 Mutations* | Not Reported |
| BTC 24 | 31.40% | Confirmed by 3rd Party Concordance | Not Reported |
| BTC 25 | 50% | Confirmed by 3rd Party Concordance | Not Reported |
| BTC 26 | 50% | Confirmed by 3rd Party Concordance | Not Reported |
| BTC 27 | 33.30% | Confirmed by 3rd Party Concordance | Not Reported |
| BTC 29 | 66.66% | Confirmed by 3rd Party Concordance | *FGFR2-SORBS* |
| BTC 30 | 53.80% | Confirmed by 3rd Party Concordance | *FGFR3 AMP* |
| BTC 33 | 100% | Confirmed by 3rd Party Concordance | Not Reported |
| BTC 34 | 20% | Confirmed by 3rd Party Concordance | *ANK-BRAF* |
| BTC 36 | 50% | Confirmed by 3rd Party Concordance | Not Reported |
| BTC 37 | 83.30% | Confirmed by 3rd Party Concordance | *FGFR1 amp* |
| BTC 39 | confirmed by TIER1 mutations | Confirmed by Tier 1 Mutations* | Not Reported |
| BTC 43 | 60% | Confirmed by 3rd Party Concordance | *FGFR1 amp* |
| **'Tier 1 Mutations' applies to samples for which internal whole exome sequencing was performed and shows the abbreviations for which mutations observed in oncoKB cancer-associated genes AND which are observed in either the MC3 or GENIE datasets with a frequency greater than or equal to one.* | | | |
|  |  |  |  |
|  |  |  |  |
