# Supplementary material for: Ex Vivo Tumor-Derived Organoid Pharmacotyping Identifies Personalized Therapeutic Options for Patients with Biliary Tract Cancer
Source: Cancer Res Commun. 2026 Aug 31;6(8):2026–38. doi: 10.1158/2767-9764.CRC-25-0792 (PMC13528702; doi:10.1158/2767-9764.CRC-25-0792)
Supplement: Supplementary file 4 [file crc-25-0792_table_s3_suppst3.docx]

**Supplementary Table 3. Cholangiocarcinoma genomic landscape from The Cancer Genome Atlas (TCGA) and comparison to this BTC cohort.** Shown are mutations, structural variants, or copy number alterations identified at a frequency ≥ 2.9%.
